# Adverse reproductive health outcomes in a cohort of young women with breast cancer exposed to systemic treatments

**DOI:** 10.1186/s13048-019-0581-6

**Published:** 2019-10-31

**Authors:** Cristina Silva, Ana Cristina Ribeiro Rama, Sérgio Reis Soares, Mariana Moura-Ramos, Teresa Almeida-Santos

**Affiliations:** 1 Pharmacology Department, Faculty of Pharmacy, University of Coimbra, Coimbra, Portugal; 2 Pharmaceutical Services, Centro Hospitalar e Universitário de Coimbra (CHUC), EPE, Coimbra, Portugal; 3 Institute for Clinical and Biomedical Research, Faculty of Medicine, University of Coimbra, Coimbra, Portugal; 4 IVI Clinic, Lisbon, Portugal; 5 Reproductive Medicine Unit, Centro Hospitalar e Universitário de Coimbra (CHUC), EPE, Coimbra, Portugal; 6 Center for Research in Neuropsychology and Cognitive and Behavioural Intervention, Coimbra, Portugal; 7 Faculty of Medicine, University of Coimbra, Coimbra, Portugal; 8 Center for Neuroscience and Cell Biology, University of Coimbra, Coimbra, Portugal

**Keywords:** Breast cancer, Chemotherapy, Ovarian reserve, Anti-mullerian hormone, Infertility, Oncofertility, Premature ovarian insufficiency, Fertility preservation

## Abstract

**Background:**

Breast cancer is the most common cancer in young women. Fortunately current survival rates of BC are significant which makes future fertility very important for quality of life of BC survivors. Chemotherapy carries a significant risk of infertility in BC patients so it is important to support fertility preservation decisions in premenopausal women. Amenorrhea has long been used as a surrogate marker of infertility in cancer patients but more reliable ovarian reserve (OR) markers are available. This study aimed to prospectively measure levels of OR in a cohort of young women with breast cancer exposed to chemotherapy, to identify adverse reproductive health outcomes in this population and to assess the influence of patient and treatment-related factors in those outcomes.

**Methods:**

This prospective observational study included premenopausal women with breast cancer aged 18–40 years at diagnosis and proposed for (neo) adjuvant chemotherapy. Patients were evaluated before, during and a minimum of 9 months after the end of chemotherapy. Reproductive health outcomes: menses, hormonal and ultrasound OR markers, recovery of ovarian function and Premature Ovarian Insufficiency (POI).

**Results:**

A total of 38 patients were included (mean age 32.9 ± 3.5 years). Levels of OR significantly decreased during the study. At the last follow up, 35 patients had AMH below the expected values for age; eight presented postmenopausal FSH; ten had not recovered their ovarian function and five met the defined criteria for POI. Age and baseline AMH were positively correlated with AMH at the last follow-up. AMH levels were higher in the group of patients treated with trastuzumab and lower in those under hormonal therapy, at the last follow-up.

**Conclusions:**

Significant effects of systemic treatments on several reproductive outcomes and a strong relation of those outcomes with patient’s age and baseline level of AMH were observed. Our results point to a possible lower gonadotoxicity when treatment includes targeted therapy with trastuzumab. Also, this investigation highlights the lack of reliable OR markers in women under hormonal therapy.

## Background

Breast cancer (BC) is the most frequent cancer among women, with an estimated 1.67 million new diagnoses in 2012 [1]. Although most cases are diagnosed in women older than 40 years of age, BC is also the leading type of cancer in younger women, with an incidence around 6.6%, being also the most lethal [2, 3]. Still, in women with BC under the age of 40, survival rates range from 72 to 85% [4, 5]. Young BC women are often treated with chemotherapy (CT) regimens that include cyclophosphamide, anthracyclines and a taxane. Other systemic therapies like targeted therapy (TT) and hormonal therapy (HT) are also frequently used, sequentially or in combination with CT. However, much is still to be known about the effects of specific combinations of systemic therapies for BC on fertility. While cyclophosphamide and anthracyclines are recognized gonadotoxics, it is not definitely established if the addition of a taxane contributes to the gonadotoxicity of CT [6]. As for TT agents, data is limited although some clinical studies do not indicate ovarian toxicity [7, 8] and HT extends treatments for up to 10 years, further narrowing the reproductive window of BC patients.

In this context, the assessment of the risk of infertility in women with BC and their reproductive counselling can be challenging tasks. There is a clear need to prospectively collect data on the reproductive health outcomes of BC patients exposed to modern systemic therapies, using reliable fertility markers, to support an informed and shared decision on fertility preservation (FP). Female reproductive potential is mainly dictated by the ovarian reserve (OR) which can be estimated through surrogate markers. Amenorrhea is currently known to be a poor and late marker of damaged ovarian function [9]. The Anti-Mullerian Hormone (AMH), produced by the granulosa cells of growing follicles and the Antral Follicle Count (AFC), are highly inter-correlated measures and currently recognized as the most specific OR markers [10–12].

The aims of this study were to prospectively measure levels of OR markers in a cohort of young women with BC exposed to chemotherapy, associated or not to other systemic treatments; to identify adverse reproductive health outcomes in this population; and to assess the influence of patient and treatment-related factors in those outcomes.

## Methods

### Patients and study design

**Fig. 1:**
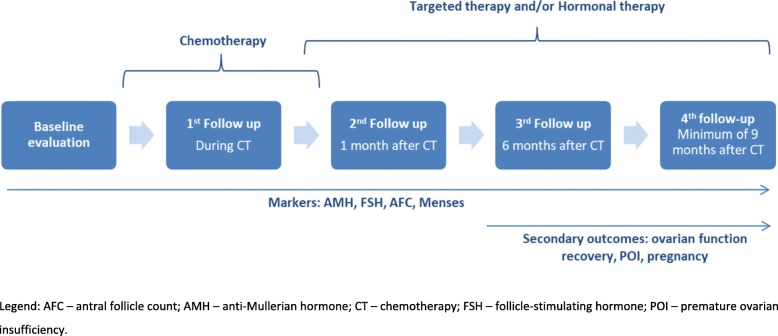
Schematic representation of the study design

This prospective observational study was conducted at the *Center for Fertility Preservation* (CFP) of the Coimbra Hospital and University Centre (CHUC, EPE). Patients included were premenopausal women with BC, aged 18–40 years at the time of diagnosis and proposed for (neo) adjuvant CT. Exclusion criteria were metastatic BC, pregnancy, levels of AMH below the quantification limit or history of previous gonadotoxic chemo/radiotherapy. Women with BC that were scheduled for a first consultation for FP counselling in the CFP were invited to participate. Recruitment took place between July 2014 and September 2016 and all participants signed an informed consent. The study received approval by the institutional ethics committee and the Portuguese Data Protection Authority. Hormonal (Follicle-Stimulating Hormone, FSH, and AMH) and ultrasound (AFC) markers of OR were assessed at several time points before, during and after CT (Fig. 1). Demographic, reproductive and clinical data were collected at recruitment (by interview and review of clinical records) and updated at subsequent appointments during and after CT.

### Reproductive health outcomes

#### Menses and ovarian reserve markers

Self-reported menstrual data was collected at the time of recruitment and updated at subsequent appointments. Amenorrhea was defined as the absence of menstrual periods and oligomenorrhea as menstrual periods occurring at intervals of more than 35 days.

Blood samples for hormonal assays were drawn regardless of the phase of the menstrual cycle. All samples were centrally analysed for AMH and FSH levels at the Clinical Pathology Department of CHUC, EPE. AMH was measured by the UltraSensitive AMH ELISA assay kit (Ansh Labs) with a Limit of Quantification (LoQ) of 0.06 ng/mL. FSH was measured by the ADVIA Centaur® FSH immunoassay, with a LoQ of 0.3 mIU/mL.

Antral follicle count (AFC) by intravaginal ultrasound was performed by experienced gynaecologists, following published recommendations [13] but regardless of the phase of the menstrual cycle. AFC was not performed in participants were under ovarian suppression.

#### Recovery of ovarian function

Recovery of ovarian function after CT was defined as: 1) return of menses *and* recovery of at least one of the measures of OR (FSH level ≤ 25 mIU/mL *or* AMH level ≥ baseline level/expected median level for age *or* AFC ≥ baseline level/ expected median count for age) or 2) the occurrence of pregnancy. The expected AMH levels and AFC according to age were set based on median results obtained by *Seifer* [14] and *Almog* [15], correspondingly.

This outcome was not assessed in women with premenopausal FSH levels that were exposed to some form of HT, as published data is not conclusive on the influence of tamoxifen and GnRH agonists on hormonal levels [16–24].

#### Premature ovarian insufficiency

According to the recommendations from the *European Society of Human Reproduction and Embryology* [25], POI was defined as the occurrence of oligo/amenorrhea for at least 4 months and elevated FSH serum levels (> 25 IU/L) on two occasions more than 4 weeks apart, after CT. Amenorrheic patients under ovarian suppression were not evaluated for this outcome.

### Statistical analysis

The analysis was performed with the software Statistical Package for Social Sciences (SPSS) version 21.

Non-parametric tests were used due to the small sample size and deviation from normality of most variables. Spearman’s Rho (*ρ*) was used to test the association between variables. *Mann- Whitney* and *Kruskall-Wallis* tests were used to compare 2 and 3 or more groups, respectively. Paired sample analysis was conducted using the Wilcoxon signed-rank test. The significance level was set at 0.05. All measurements of AMH below the limit of quantification (LoQ = 0.06 ng/mL) were assigned the value of 0.06 ng/mL.

## Results

### Patients’ characteristics and cancer treatments

**Fig. 2:**
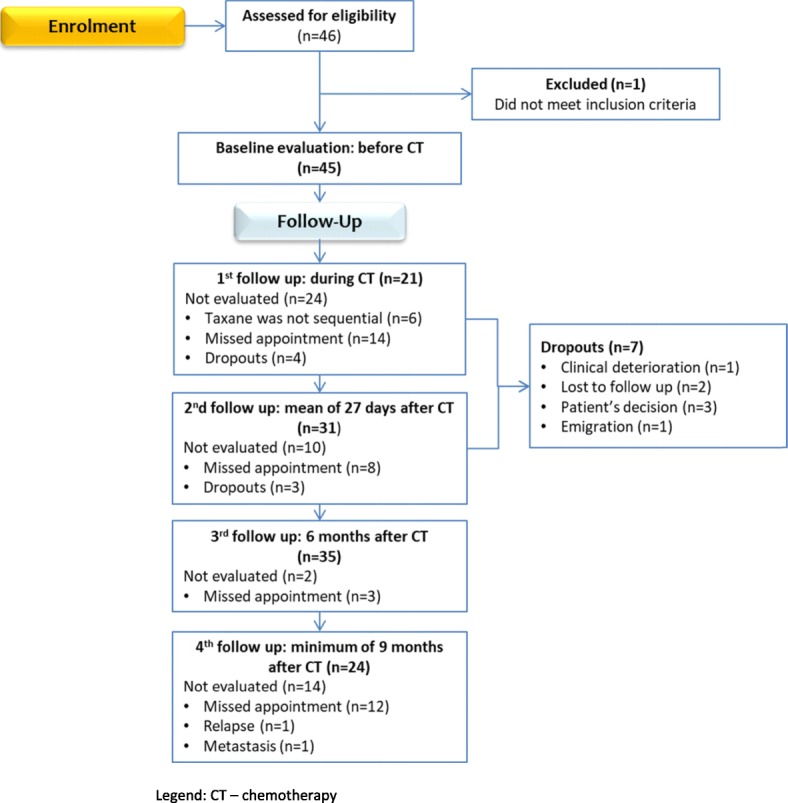
Flow diagram of participation in the study

A total of 46 women were recruited (Fig. 2). Median age of participants at study inclusion was of 33 years (mean 32.9 ± 3.5 years; min 25-max 39). The last follow-up occurred at a mean of 18 months (range 6–35 months) after the end of CT and a mean of 2 years (range 1–3 years) after recruitment.

Demographic, clinical and fertility preservation data for the 38 participants are detailed in Table 1. Most women (32/38; 84%) received an anthracycline-based chemotherapy regimen followed by a taxane. CT duration varied from 12 to 24 weeks with a median of 18 weeks.

**Table 1:** Baseline demographic, obstetric, fertility preservation and treatment characteristics of the study participants (*n* = 38)

Characteristics	
Mean age [median] (years)	32.9 ± 3.5 [33.0]
Married/with partner; n (%)	28 (74)
Childless; n (%)	25 (66)
Overweight / Obese; n (%)	9 (24)
Former/current smokers; n (%)	11 (29)
Fertility preservation; n (%)	
No	9 (24)
Yes	29 (76)
*Embryo CP*	*2*
*Oocyte CP*	*25*
*Ovarian tissue CP*	*2*
*GnRH agonist*	*2*
Tumour biology; n (%)	
HR-positive	15 (39)
HER_2_-positive	5 (13)
Triple negative	9 (24)
Triple positive	9 (24)
Type of systemic treatments; n (%)	
CT only	8 (21)
CT + HT	16 (42)
CT + TT	5 (13)
CT + TT + HT	9 (24)
CT type; n (%)	
Neoadjuvant	21 (55)
Adjuvant	17 (45)
Type of CT regimen; n (%)	
Anthracycline-based (FEC/AC/EC) + sequential Paclitaxel/Docetaxel	32 (84)
Taxane-based	6 (16)
Type of HT; n (%) (*n* = 25)	
Tamoxifen only	7 (28)
Tamoxifen + GnRHa	10 (40)
Aromatase inhibitor only	1 (4)
Aromatase inhibitor + GnRHa	5 (20)
GnRHa only	2 (8)

Data are presented as mean ± SD or *n*

*BMI* Body Mass Index, *CT* Chemotherapy, *HC* Hormonal contraception

*HER2* Human Epidermal growth factor Receptor-type 2, *HR* hormonal receptors

*HT* hormonal therapy, *GnRHa* Gonadotropin-Releasing Hormone agonist

*TT* targeted therapy

Twenty nine participants performed fertility preservation through the use of one or more techniques, mainly oocyte cryopreservation (*n* = 25). Two women cryopreserved embryos, two underwent ovarian tissue cryopreservation and two were prescribed a GnRH agonist. At the end of the study none of the participants had attempted to get pregnant through the use of their cryopreserved cells or ovarian tissue.

### Reproductive health outcomes

#### Menses

All patients reported regular menses at the time of recruitment. At the last follow-up, 25 in a total of 35 patients (71%) reported amenorrhea or oligomenorrhea. Most of these amenorrheic patients were under ovarian suppression (*n* = 16). Among the ten patients reporting menses, seven (7/10; 70%) presented decreased OR (AMH levels below the normal for their age and/or FSH levels > 25 mIU/mL).

#### Ovarian reserve markers

##### Evolution of ovarian reserve markers

Table 2 presents the mean levels of AMH, AFC and FSH measured at baseline, during CT, at a mean of 27 days after the end of CT and at each patient’s last available follow-up (mean of 18 months after the end of CT). At baseline, levels of AMH and AFC were strongly inter-correlated (*ρ* = 0.656; *p* < 0.001) and negatively correlated with patient’s age (AMH, *ρ* = − 0.41, *p* = 0.01; AFC, *ρ* = − 0.37, *p* = 0.028).

**Table 2:** Mean and median levels of ovarian reserve markers (AMH, AFC and FSH) measured at baseline, during CT, 1 month and at a mean of 18 months after the end of CT

	Baseline	During CT	1 month after CT	Last available follow-up
AMH ng/mL Mean ± SD (median)	3.07 ± 2.95 (2.20) *n* = 38	0.30 ± 0.50 (0.06) *n* = 21	0.15 ± 0.46^1^ (0.06) *n* = 30	0.32 ± 0.68^1,3^ (0.06) *n* = 34
AFC Mean ± SD (median)	10.6 ± 5.2 (9.0) *n* = 35	4.1 ± 3.5 (4.0) *n* = 19	3.4 ± 6.8^2^ (1.0) *n* = 25	2.2 ± 3.0^1^ (2.0) *n* = 21
FSH mIU/mL Mean ± SD (median)	7.1 ± 5.6 (5.2) *n* = 36	21.2 ± 24.2 (9.1) *n* = 21	64.3 ± 47.8^1^ (64) *n* = 28	21.4 ± 36.9^4^ (6.4) *n* = 35

*AFC* antral follicle count, *AMH* anti-Mullerian hormone, *CT* chemotherapy, *FSH* follicle-stimulating hormone

*SD* standard deviation

^1^ as compared to baseline, p<0.001; ^2^ as compared to baseline, *p* = 0.001; ^3^ as compared to the previous follow-up, *p* = 0.03;

^4^as compared to the previous follow-up, p<0.001

***Anti-Mullerian hormone (AMH)***. The levels of AMH significantly decreased from baseline to the post-CT follow-ups, both at 1 month (z = − 4.78, *p*<0.001) and at a mean of 18 months after CT (z = − 4.9; *p*<0.001). However, a significant increase of AMH levels between these two last follow-ups was noticed (z = − 2.9, *p* = 0.003). The number of patients with AMH levels below the LoQ increased from 12 during CT to 28 at 1 month after CT, and then decreased to 21, at the last follow-up. Still, at the end of the study, 30 patients (30/35; 86%) had AMH levels below the expected values for age and only one had recovered to baseline levels.

***Antral follicle count (AFC)***. At baseline, 35 patients performed AFC and 16 presented normal counts considering the expected values for their age (16/35; 46%). The number of performed counts was reduced to 21, at the last available follow-up, mainly due to ovarian suppression. Mean AFC progressively and significantly decreased until the last follow-up (z = − 3.9, *p* < 0.001). No patient recovered to their initial AFC and only one presented an AFC above the normal median value for her age, at her last follow-up (28 months after the end of CT).

***Follicle-stimulating hormone*** .Although a high variability was observed, the levels of FSH significantly increased at the follow-up 1 month after CT (*n* = 28; z = − 4.2, *p* < 0.001) although a significant decrease was found at last follow-up (z = − 3.8, *p* < 0.001). Still, at the last follow-up, eight participants (8/30; 27%) had levels consistent with menopausal status (> 25 mIU/mL). No significant correlations were found between FSH levels at the last follow-up and time to follow-up (*ρ* = 0.035; *p* = 0.881), baseline FSH (*ρ* = 0.235; *p* = 0.181) or patients’ age (*ρ* = 0.125; *p* = 0.474).

##### Ovarian reserve markers at the last follow-up

**Fig. 3:**
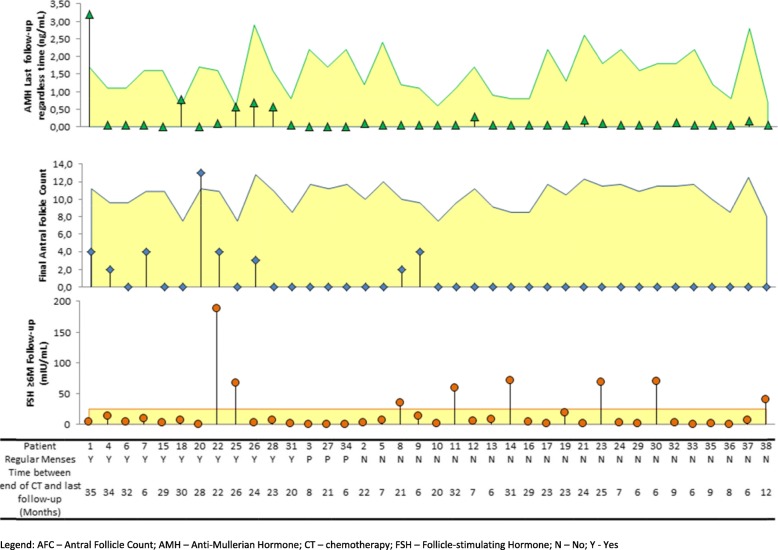
Patients’ levels of OR markers at the last follow-up, ordered according to menstruation status (Y/N), and their comparison with the corresponding expected levels for age (AMH, AFC) or the cut-off value of 25 mIU/mL (FSH)

AMH and AFC levels at the last follow-up were positively and significantly inter-correlated (*n* = 21; *ρ* = 0,429; *p* = 0.05). Figure 3 gives a general insight on how the levels of OR markers compared with the corresponding levels expected according to patient’s age (for AMH, AFC) or the defined cut-off value (for FSH). It is clear that most patients presented lower than expected values of AMH (30/35; 86%) and AFC (20/21; 95%). Figure 3 also highlights the fact that the levels of AMH and AFC were lower than expected in many of the patients who recovered regular menses.

#### Recovery of ovarian function (ROF)

At their last available follow-up, ten women (ages of 30–39 years) had not recovered their ovarian function (10/19; 53%). Two of these women had not preserved fertility before CT. Five of them reported amenorrhea, three irregular menses and all presented AMH levels below the LoQ and/or FSH levels consistent with ovarian failure. In a total of 19 patients (*n* = 19), ROF was not evaluated as they were under HT and presented premenopausal levels of FSH.

In contrast, nine women met the criteria for ovarian function recovery at their last follow-up (9/19; 47%). They were aged 25 to 38 years at recruitment and most presented normal/high levels of OR at baseline. Four of these women got pregnant at some point after CT. Two of them were under therapy with trastuzumab at the time of pregnancy and reported either spontaneous or medically-induced abortions. Pregnancy outcome was not known in the other two patients. Three of the woman who got pregnant had normal/high levels of OR markers at baseline. The only patient with low OR at baseline was treated with a taxane-only CT regimen.

#### Premature ovarian insufficiency (POI)

A total of five patients met the defined criteria for POI (5/22; 23%), an outcome that was only assessed in patients who were not under ovarian suppression with GnRHa. Patients with POI had ages between 32 and 39 years at recruitment. Their levels of AMH remained below the LoQ and FSH was persistently high. One of them had not preserved her fertility before CT.

#### Overall adverse reproductive health outcomes

A summary of the various adverse reproductive health outcomes identified in this cohort of young BC women, at a mean of 18 months after CT, is presented in Table 3.

**Table 3:** Percentage of patients in which the several adverse reproductive health outcomes were identified, at the last follow-up

Adverse outcome	Oligo/ amenorrhea	AMH < LoQ	AMH < expected for age	AFC < expected for age	FSH > 25 mIU/mL	No recovery of ovarian function	Premature Ovarian Insufficiency
Percentage of patients presenting the outcome (total number of patients evaluated)	71% (35)	55% (38)	86% (35)	95% (21)	25% (36)	53% (19)	23% (22)

*AFC* Antral Follicle Count, *AMH* Anti-Mullerian Hormone, *FSH* Follicle-Stimulating Hormone, *LoQ* Limit of Quantification

### Patient-related factors and adverse reproductive health outcomes

#### Age at recruitment

Age at recruitment was negatively correlated with the levels of AMH at the last follow-up (*n* = 35; *ρ* = − 0.377; *p* = 0.026). Moreover, AMH levels at the last follow-up were significantly higher in the group of younger patients (*n* = 16; age < 33 years) as compared to those aged 33 or more years (*n* = 19) and this difference was significant (U = 70.5; *p* = 0.002). In opposition, no significant correlation of patients’ age with final AFC was found (*n* = 21; *ρ* = − 0.183; *p* = 0.427).

In this cohort of women with BC, age at recruitment was significantly lower in patients who recovered ovarian function as compared to those who did not recover (U = 20; *p* = 0.038). However, in the subgroups of women with/without POI and women reporting/not regular menses, age at recruitment was not significantly different.

#### Baseline ovarian reserve

A significant positive correlation between AMH levels at baseline and at the last follow-up was found (*n* = 35; *ρ* = 0.517; *p* = 0.001). Levels of AMH at the last follow-up were also higher in the subgroup of patients with a baseline AMH above 2.2 ng/mL (the median for the cohort). In contrast, no significant correlation was found between baseline and final AFC (*n* = 20; *ρ* = 0.275; *p* = 0.241).

Baseline AMH levels also influenced the likelihood of recovering ovarian function, experiencing POI or reaching postmenopausal FSH levels: baseline AMH levels were significantly higher in the subgroup of women who recovered ovarian function (*n* = 19; U = 19.5; *p* = 0.037) and significantly lower in patients with POI or menopausal FSH levels at the last follow-up (*n* = 22; U = 12, *p* = 0.017 and *n* = 19; U = 15, *p* = 0.022, respectively). Significant differences in AFC at baseline were only found between the groups of patients with/without POI at the last follow-up (U = 9.5; *p* = 0.032).

### Treatment-related factors and adverse reproductive health outcomes

#### Chemotherapy regimens

No significant differences were found when comparing OR levels at the last follow-up in specific subgroups of CT (FEC-taxane, EC/AC-taxane, taxane without anthracycline, taxane-only and others). Nevertheless, in the subgroup of patients treated with sequential taxane (*n* = 17), levels of AMH were significantly lower after exposure to taxane (z = − 2.2; *p* = 0.028). The only CT regimen in which no negative outcome was identified was the *paclitaxel-only* regimen (*n* = 2). In this group, one patient got pregnant and the other recovered regular menses and normal expected OR levels.

No significant correlations were found between the number of weeks of exposure to CT and the levels of AMH or AFC at the last follow-up.

#### Treatment combinations, exposure to trastuzumab and to hormonal therapy

AMH levels at the last follow-up were compared in the groups treated with the four different combinations of systemic treatments. AMH was significantly higher in the group treated with CT + TT (*n* = 3) as compared to the groups exposed to CT only (*n* = 7) (U = 2; *p* = 0.046) and to CT + HT (*n* = 16) (U = 0; *p* = 0.002). In accordance with these results, we also found that AMH levels at the last follow-up were higher in all patients treated with trastuzumab (*n* = 12) as compared to those not exposed (U = 84.5; *p* = 0.036). No significant differences in mean age, baseline AMH levels or time to follow-up were found between these two groups.

Patients that were under treatment with any type of HT at the last follow-up exhibited significantly lower AMH (U = 88; *p* = 0.05) and FSH (U = 83; *p* = 0.056), although these groups were not different regarding their baseline age or AMH levels. When comparing the same OR markers in patients exposed/not exposed to GnRHa at the last follow-up, no significant differences in AMH levels were found, although FSH remained lower in those under ovarian suppression (U = 57; *p* = 0.002).

## Discussion

### Adverse reproductive health outcomes

It is of concern that more than half of the young premenopausal BC patients in this cohort did not recover ovarian function (10/19; 53%), at a mean of 18 months after the end of CT. Additionally, we have identified POI in five women, which represent 23% (5/22) of those who could be evaluated for this outcome. A diagnosis of POI has a significant negative impact on the psychological wellbeing and quality of life as women may also experience genitourinary symptoms and present a reduced bone mineral density and an increased cardiovascular risk [25]. A considerable number of patients in our study were not evaluated for recovery or POI due to exposure to HT, so it is likely that the incidence of these adverse outcomes would increase with a longer follow-up. Other studies have shown that ovarian function recovery may occur up to 2 years after the end of CT in women with BC [26–28], so we anticipate that most women in this cohort have now a very low, if any, chance of recovery. A similar prevalence of POI in young BC patients was observed for the control group of the OPTION trial (6/30; 20%) [29]. Our results are also in accordance with the subsequent data analysis of this trial by Anderson and colleagues [30], that concluded that women who developed POI had lower pre-treatment AMH concentrations than those who did not.

At the last follow-up, other adverse reproductive health outcomes are noteworthy: i) only one woman recovered her baseline AMH levels, ii) only five presented AMH levels considered normal according to age and iii) serum AMH was below the LoQ in 60% of the participants. Moreover, ten patients presented altered OR markers despite the recovery of regular menses, in line with other published studies [31, 32]. Nevertheless, four women became pregnant during the course of the study. Similarly to previous studies [19, 33], pregnancy occurred regardless of the low levels of OR.

### Patient-related factors

In our very young cohort that may best represent the group of BC patients who engage in fertility counselling, age and baseline AMH were positively correlated with AMH levels at the last follow-up and the participants who recovered ovarian function were younger. Our results confirm AMH as the most sensitive marker of ovarian damage in BC patients exposed to CT and reinforce the potential usefulness of AMH as a predictor of ovarian function after CT, in line with other studies where pre-CT level of AMH was associated with the occurrence of amenorrhea [34] or with post-CT AMH levels [19, 20].

### Treatment-related factors

The addition of taxanes to anthracycline-based regimens has been associated with an increased negative impact of CT on fertility in several published clinical studies [7, 35, 36] and meta-analysis [37], despite a few studies reporting opposite results [28, 38]. In our cohort, we found significant differences in the levels of AMH before and after the administration of the taxane.

One of the possible ways to overcome the negative effects of CT treatments in fertility is to select less gonadotoxic CT regimens. In our study, no adverse reproductive health outcome was observed in the two participants treated with the weekly paclitaxel regimen. These results are in accordance with those from the APT trial [39], where the weekly paclitaxel regimen seemed to have caused a less pronounced gonadotoxic effect. Further prospective controlled studies are needed to test this theory.

An interesting and somehow unexpected result of our investigation was the significant higher AMH levels in patients treated with trastuzumab. Trastuzumab is a monoclonal antibody that targets HER2-expressing tumour cells, and pre-clinical reproductive studies showed no evidence of impaired fertility [40]. Our results concur for the lack of gonadotoxicity that was already pointed by other clinical studies [7, 41] and add further data to the hypothesis of trastuzumab as a protector of ovarian vasculature from CT-induced damage [8]. In this study by Ben-Aharon and colleagues, a milder decrease in ovarian blood flow was seen in patients treated with trastuzumab as compared to those treated with CT only. The relevance of our observation is also supported by the results of a recently published cross-sectional analysis, where exposure to trastuzumab was associated with increased AMH in BC survivors with normal menstrual cycles (*n* = 25) [42]. Moreover, this finding corroborates the previously mentioned outcomes of the APT trial [39]. There is a clear need for further investigations to ascertain this protective effect and to clarify the potential mechanism of action of trastuzumab.

In our study, we also found evidence of the influence of HT in the levels of OR markers: patients under therapy with tamoxifen, aromatase inhibitor and/or GnRHa at the last follow-up exhibited significantly lower levels of AMH and FSH, despite no differences in their baseline ages and AMH levels were found. So, premenopausal FSH levels in BC patients under HT may be falsely reassuring [43]. When the isolated effect of ovarian suppression with GnRHa was investigated, only FSH levels remained different. Previous studies have also reported that patients under treatment with tamoxifen and/or a GnRHa may experience reduced FSH levels [22, 33, 44]. Regarding the influence of GnRHa on AMH, some authors believe they have no direct effect on OR, due to the absence of FSH, LH, or GnRH receptors in primordial follicles [45] but others have reported reduced AMH levels in patients under ovarian suppression [20, 24]. Further investigation is needed to confirm AMH as a reliable marker in this setting.

### Limitations

This small cohort study may have lacked statistical power to detect differences between treatment combinations and different CT regimens. In view of this limitation, we have decided not to develop predictive models or conduct multivariate analysis.

Our results might also have been influenced by the low baseline OR levels seen in some participants, although the median AMH level of 2.2 ng/mL was similar to levels reported in other studies in younger BC patients [26].

Two participants in our study performed ovarian tissue cryopreservation (OTCP) which may have further contributed to reduce their OR. According to the recent study by Lantsberg et al. [46], that included 203 participants who performed OTCP, women with a breast cancer diagnosis and those under 30 years of age are more likely to conceive naturally and consequently less likely to need ovarian tissue transplantation. We were not able to confirm the influence of this FP technique on the levels of OR markers but it is important to notice that the two women who performed OTCP were over the age of 30 at diagnosis and presented lower than expected AMH level at baseline.

Other major limitation is the fact that a very significant number of participants (*n* = 20) was still under the influence of HT at the last follow-up, which restricted the assessment of some of the outcomes. Nevertheless, this limitation also occurs in clinical practice: many BC patients remain in treatment with HT for several years and the identification of reliable markers to assess their menopausal status is still an unsolved issue [47, 48].

## Conclusions

Our study in young women with BC revealed significant effects of systemic treatments on several reproductive outcomes and confirmed their strong relation with patient’s age and baseline level of AMH. We have confirmed AMH as the most sensitive marker of OR in young premenopausal women with BC and its effectiveness as a predictor of ovarian function recovery and occurrence of POI.

The results of this study point to the possibility of a lower gonadotoxicity when patients are treated with less complex CT regimens or when treatment includes targeted therapy with trastuzumab. Also, this investigation highlights the lack of reliable OR markers in women with BC under treatment with HT and the consequent risk of undetected ovarian failure in this population.

Overall, our results strongly emphasize the relevance of pre-treatment counselling regarding infertility risks and fertility preservation for all premenopausal BC patients, with a special emphasis on older patients or those with low baseline OR.

## Data Availability

The datasets collected and/or analyzed during the current study are available from the corresponding author on reasonable request.

## Abbreviations

AFC: Antral follicle count
AMH: Anti-Mullerian hormone
BC: Breast cancer
CFP: Center of Fertility Preservation
CT: Chemotherapy
FP: Fertility preservation
FSH: Follicle-stimulating hormone
GnRHa: Gonadotropin-releasing hormone agonist
HT: Hormonal therapy
LoQ: Limit of quantification
OR: Ovarian reserve
POI: Premature ovarian insufficiency
